# Epidemiology, Quality and Reporting Characteristics of Systematic Reviews of Traditional Chinese Medicine Interventions Published in Chinese Journals

**DOI:** 10.1371/journal.pone.0020185

**Published:** 2011-05-25

**Authors:** Bin Ma, Jiwu Guo, Guoqing Qi, Haimin Li, Jiye Peng, Yulong Zhang, Yanqin Ding, Kehu Yang

**Affiliations:** 1 Evidence-Based Medicine Center, Institute of Traditional Chinese and Western Medicine, School of Basic Medical Sciences, Lanzhou University, Lanzhou, Gansu, China; 2 Second School of Clinical Medicine of Lanzhou University, Lanzhou, Gansu, China; 3 The Library of Lanzhou University, Lanzhou, Gansu, China; Alberta Research Centre for Health Evidence, University of Alberta, Canada

## Abstract

**Background:**

Systematic reviews (SRs) of TCM have become increasingly popular in China and have been published in large numbers. This review provides the first examination of epidemiological characteristics of these SRs as well as compliance with the PRISMA and AMSTAR guidelines.

**Objectives:**

To examine epidemiological and reporting characteristics as well as methodological quality of SRs of TCM published in Chinese journals.

**Methods:**

Four Chinese databases were searched (CBM, CSJD, CJFD and Wanfang Database) for SRs of TCM, from inception through Dec 2009. Data were extracted into Excel spreadsheets. The PRISMA and AMSTAR checklists were used to assess reporting characteristics and methodological quality, respectively.

**Results:**

A total of 369 SRs were identified, most (97.6%) of which used the terms systematic review or meta-analysis in the title. None of the reviews had been updated. Half (49.8%) were written by clinicians and nearly half (47.7%) were reported in specialty journals. The impact factors of 45.8% of the journals published in were zero. The most commonly treated conditions were diseases of the circulatory and digestive disease. Funding sources were not reported for any reviews. Most (68.8%) reported information about quality assessment, while less than half (43.6%) reported assessing for publication bias. Statistical mistakes appeared in one-third (29.3%) of reviews and most (91.9%) did not report on conflict of interest.

**Conclusions:**

While many SRs of TCM interventions have been published in Chinese journals, the quality of these reviews is troubling. As a potential key source of information for clinicians and researchers, not only were many of these reviews incomplete, some contained mistakes or were misleading. Focusing on improving the quality of SRs of TCM, rather than continuing to publish them in great quantity, is urgently needed in order to increase the value of these studies.

## Introduction

The first systematic review addressing the effect of traditional Chinese medicine (TCM) that was published in a Chinese journal may be sourced back to Chen et al. in 1999 [1]. Since then, systematic reviews of TCM have become been increasingly published in China, and now with a large number. Given the implications of systematic reviews to policy making and clinical practice, achieving highest possible quality in design, conduct, analysis, and reporting is of paramount importance [2].

In the last decade, a few studies have examined the reporting of systematic reviews, and found that the quality of reporting was generally poor [3], [4], [5]. Three studies, using the Quality of Reporting of Meta-analyses (QUOROM) checklist, examined the quality of reporting of reviews of traditional Chinese medicine, and concluded with similar findings [6], [7], [8] Nevertheless, they were inherent with limitations, including the failure to report details about epidemiological characteristics of included reviews, and the failure to address methodological quality of those reviews.

In 2009, the newer standard of reporting systematic review, the Preferred Reporting Items for Systematic Reviews and Meta-Analyses (PRISMA) was released to replace the QUOROM for guiding the review reporting [9]. Earlier than that, an instrument, assisting the assessment of methodological quality of systematic reviews, Assessment of Multiple Systematic Reviews (AMSTAR) [10] was published in 2007. This instrument, developed on the biasis of the Oxman-Guyatt Overview Quality Assessment Questionnaire (OQAQ) [11] and Sack's Quality Assessment Checklist [12] is considered a validated tool in assessing the methodological quality of systematic reviews, and receives recognition from international agencies, including The Canadian Agency for Drugs and Technologies in Health (CADTH) [13].

Our study therefore aims to address the limitations of published studies, by assessing epidemiological and reporting characteristics, and methodological quality of systematic reviews of traditional Chinese medicine. We will particularly employ the new instruments for assessing the quality of reporting and methodological quality of those reviews.

## Methods

### Inclusion Criteria

We included all systematic reviews about TCM published in Chinese Journals. TCM interventions may have included herbal medicine, acupuncture/acupressure, moxibustion, Tuina massage, food therapy, and physical exercise such as tai chi and shadow boxing. TCM interventions may have been administered alone or in combination with conventional western medicine. We included publications described as systematic reviews or those that provided an overview of evidence from multiple studies and where authors described their methods in explicit detail.

### Search Strategy(Text S1)

Four Chinese databases (Chinese Biomedicine Literature Database (CBM), Chinese Scientific Journal Full-text Database (CSJD), Chinese Journal Full-text Database (CJFD), and Wanfang Database) were searched from inception through Dec 2009. The search terms included “Systematic Review”, “Meta-analysis”, “Traditional Chinese Medicine” and “Chinese herbs” etc.

### Screening

Two researchers independently screened the titles and abstracts of identified studies. One reviewer subsequently screened the full text articles of potentially included studies (Jiwu GUO) while a second reviewer independently screened a 20% random sample (Bin MA). Disagreement was resolved by discussion.

### Data Collection and Analysis

Variables extracted included publication and reporting characteristics as well as items from the PRISMA and AMSTAR checklists. Reviews were classified according to their TCM focus as Herbal (e.g. bulk herbs, decoctions, pills) or Non-herbal (e.g. acupuncture, Tuina). Conditions studied were classified using the International Classification of Diseases (ICD-10). Data was collected using a standardized form and summarized using descriptive statistics (frequency, median, interquartile range (IQR)). Analyses were performed using Excel (version Microsoft Excel 2003; http://office.microsoft.com/zh-cn/) and SPSS (version 13.0; http://www.spss.com).

## Results

### Search

**Figure 1:**
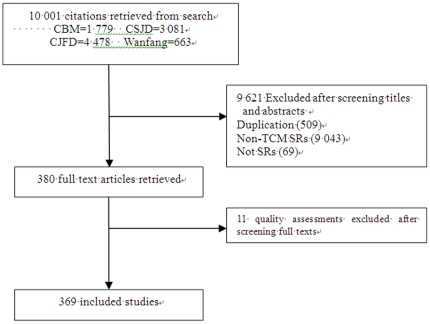
Flow chart of articles identified, included and excluded. SRs, Systematic Review.

The searches identified 10,001 records. Screening excluded 9,621 reviews due to duplication, focus on non-TCM interventions, or for not being a SR. After examination of the full texts of 380 article, a further 11 reviews were excluded because they were quality assessments of systematic reviews and meta-analyses. A total of 369 publications were included (Figure 1, Text S2).

### Epidemiological Characteristics (Table 1)

**Table 1:** Epidemiology of Systematic Reviews.

Category	Characteristic	Number (%) of n = 369
**Number of times cited**	0	170 (46.1)
	1–5	137 (37.1)
	6–10	41 (11.1)
	11–15	12 (3.3)
	>15	9 (2.4)
**Role of first author**	Clinician	184 (49.9)
	Researcher	82 (22.2)
	Graduate student	54 (14.6)
	Other	49 (13.3)
**Focus of reviews**	Chinese Herbal Interventions^a^	314 (84.3)
	Non-Herbal Interventions^b^	55 (15.7)
**Condition focused on in review (Common ICD-10^c^)**	Diseases of the circulatory system	24 (6.5)
	Diseases of the digestive system	21 (5.7)
	Diseases of the nervous system	18 (4.9)
	Diseases of the genitourinary system	17 (4.5)
	Diseases of the respiratory system	11 (3.0)
	Diseases of the blood and blood-forming organs and immune mechanism	14 (3.8)
	Diseases of the musculoskeletal system and connective tissue	9 (2.4)
	Mental and behavioural disorders	9 (2.4)
	Diseases of the skin and subcutaneous tissue	5 (1.4)
	Endocrine, nutritional and metabolic diseases	5 (1.4)
	Neoplasms	5 (1.4)
	Infectious and parasitic diseases	3 (0.8)
	Pregnancy, childbirth and the puerperium	2 (0.5)
	Diseases of the ear and mastoid process	1 (0.3)
	Diseases of the eye and adnexa	1 (0.3)
	Symptoms, signs and abnormal clinical and laboratory findings, not classified elsewhere	1 (0.3)

a Herbal interventions included bulk herbs, decoctions, pills;

b Non-herbal interventions included acupuncture and Tuina;

c Common ICD-10: International Classification of Diseases 10.

The 369 reviews were all written in Chinese and were published in 145 different Chinese Journals. Frequency of citation of each review ranged from 0 to 79; nearly half (46.1%) had not been cited and only 2.4% had been cited more than 15 times. Almost half (49.9%) of the reviews were written by clinicians. The reviews were classified as either Herbal interventions (84.3%) or Non-Herbal interventions. Non-herbal interventions included acupuncture (14.6%) and Tuina (1.1%). The most common conditions studied were diseases of the circulatory system (6.5%) and digestive system (5.7%).

### Descriptive Characteristics (Table 2)

**Table 2:** Descriptive Characteristics of Included Systematic Reviews.

Category		All SRs n = 369	Herbal SRs^a^ n = 314	Non-herbal SRs^b^ n = 55
**Number of authors, Median (IQR)**		3 (2–5)	3.0 (2–4)	4 (3–5)
**Journal type** *	General, n (%)	193 (52)	175 (91)	18 (9)
	Specialty, n (%)	176 (48)	139 (79)	37 (21)
**Indexed in CSCD** c Yes n (%)*		120 (33)	91 (76)	29 (24)
**Number of included studies**Median (IQR)*		10 (7–17)	11 (7–19)	8 (6–13)
**Number of participants in included studies** Median (IQR)		952 (562–1596)	1004 (583–1789)	890 (534–1273)
**Meta-analysis** Yes (%)		343 (93)	292 (93)	51 (93)
**Update of a previous review** n (%)		0 (0)	0 (0)	0 (0)

a Herbal interventions included bulk herbs, decoctions, pills;

b Non-herbal interventions included acupuncture and Tuina;

c CSCD: Chinese Science Citation Database;

*indicates p<0.05.

The reviews included a median of three authors (IQR: 2.0–5.0). Almost half the reviews (47.7%) were published in specialty journals although this differed significantly between reviews of herbal and non-herbal interventions (79.0% versus 21.0%, respectively (p<0.05). Only one-third (32.5%) were published in journals cited by Chinese Science Citation Database (CSCD), which again varied significantly between herbal and non-herbal interventions (p<0.05). The reviews included a median of 10.0 studies each, involving 951.5 participants. Non-herbal SRs included significantly fewer studies (p<0.05) and participants compared to herbal SRs. Meta-analysis was conducted in almost all the reviews (93.0%) and did not differ significantly between groups. None of the reviews had been updated from a previous review.

### PRISMA Checklist Assessment (Table 3)

**Table 3:** PRISMA Assessment of Reporting Characteristics.

Category	Item (Yes)	Overall, n = 369n (%)	Herbs^a^ n = 314n (%)	Non-herbs^b^ n = 55 n (%)
**TITLE**	1. Title	360 (97.6)	308 (98.1)	52 (94.6)
**ABSTRACT**	2. Structured summary	0 (0.0)	0 (0.0)	0 (0.0)
**INTRODUCTION**	3. Rationale	344 (93.2)	292 (93.0)	52 (94.6)
	4. Objectives	74 (20.1)	61 (19.4)	13 (23.6)
**METHODS**	5. Protocol and registration	0 (0.0)	0 (0.0)	0 (0.0)
	6. Eligibility criteria	214 (58.0)	185 (58.9)	29 (52.7)
	7. Information sources*	363 (72.1)	217 (69.1)	49 (89.1)
	8. Search*	208 (56.4)	165 (52.6)	43 (78.2)
	9. Study selection*	244 (66.1)	215 (68.5)	29 (52.7)
	10. Data collection process*	160 (43.4)	127 (40.5)	33 (60.0)
	11. Data items*	52 (14.1)	35 (11.2)	17 (30.9)
	12. Risk of bias in individual studies*	265 (71.8)	212 (67.5)	43 (78.2)
	13. Summary measures*	275 (74.5)	228 (72.6)	47 (85.5)
	14. Synthesis of results	143 (38.8)	126 (40.1)	17 (30.9)
	15. Risk of bias across studies	196 (53.1)	173 (55.1)	23 (41.8)
	16. Additional analyses	137 (37.1)	121 (38.5)	16 (29.1)
**RESULTS**	17. Study selection*	166 (45.0)	149 (47.5)	17 (30.9)
	18. Study characteristics	224 (60.7)	189 (60.2)	35 (63.6)
	19. Risk of bias within studies	227 (61.5)	187 (59.6)	40 (72.7)
	20. Results of individual studies	295 (80.0)	251 (79.9)	44 (80.0)
	21. Synthesis of results*	171 (46.3)	153 (48.7)	18 (32.7)
	22. Risk of bias across studies	151 (40.9)	133 (42.4)	18 (32.7)
	23. Additional analysis	78 (21.1)	71 (22.6)	7 (12.7)
**DISCUSSION**	24. Summary of evidence	0 (0.0)	0 (0.0)	0 (0.0)
	25. Limitations	346 (93.8)	296 (94.3)	50 (90.9)
	26. Conclusions	339 (91.9)	288 (91.7)	51 (92.7)
**FUNDING**	27. Funding	144 (39.0)	119 (37.9)	25 (45.5)

a Herbal interventions included bulk herbs, decoctions, pills;

b Non-herbal interventions included acupuncture and Tuina;

*indicates p<0.05.

Compliance with PRISMA checklist items ranged from 0–97.6%. Almost all reviews (97.6%) described themselves using the terms “systematic review” or “meta analysis”. Most reviews were compliant with the following checklist items: included a clear rationale, described information sources, described method used for assessing risk of bias of individual studies, stated the principle of summary measures, described the results of individual studies, discussed limitations at study and outcome level, provided a general interpretation of results, and presented an available conclusion. More than half of reviews were compliant with the following checklist items: reported eligibility criteria, presented search strategy, stated the process for selecting studies, specified any assessment of risk of bias that may affect the cumulative evidence, described characteristics of included studies, or described risk of bias within studies. Less than half of reviews were compliant with the following checklist items: described data collection process, described synthesis of results, described additional analysis, described how the studies were selected, presented synthesis of results (although this varied across reviews category), presented results of any assessment of risk of bias across studies, or described sources of funding and other support. Few studies provided a description of objectives or purpose, lists of data items, or gave results of additional analysis. None of the studies provided a structured summary, protocol or registration information, or provided a summary of results in the discussion.

### AMSTAR Checklist Assessment (Table 4)

**Table 4:** AMSTAR Assessment of Methodological Characteristics.

Category (Yes)	Overall, n = 369 n (%)	Herbs, n = 314n (%)	Non- herbal interventions, n = 55 n (%)
1. Was an ‘a priori’ design provided?	19 (5.2)	17 (5.4)	2 (3.6)
2. Was there duplicate study selection and data extraction? *	152 (41.2)	120 (38.2)	32 (58.2)
3. Was a comprehensive literature search performed? *	203 (55.0)	159 (50.6)	44 (80.0)
4. Was the status of publication (i.e. grey literature) used as an inclusion criterion?	10 (2.7)	9 (2.9)	1 (1.8)
5. Was a list of studies (included and excluded) provided?	13 (3.5)	10 (3.2)	3 (5.5)
6. Were the characteristics of the included studies provided?	221 (59.9)	182 (58.0)	39 (70.9)
7. Was the scientific quality of the included studies assessed and documented?	259 (70.2)	219 (69.8)	42 (76.4)
8. Was the scientific quality of the included studies used appropriately in formulating conclusions?	254 (68.8)	212 (67.5)	40 (72.7)
9. Were the methods used to combine the findings of studies appropriate? *	108 (29.3)	100 (31.9)	8 (14.6)
10. Was the likelihood of publication bias assessed? *	161 (43.6)	147 (46.8)	14 (25.5)
11. Was the conflict of interest stated?	30 (8.1)	27 (8.6)	3 (5.5)

a Herbal interventions included bulk herbs, decoctions, pills;

b Non-herbal interventions included acupuncture and Tuina;

*indicates p<0.05.

Compliance with AMSTAR checklist items ranged from 0–70.2%. More than half of reviews were compliant with the following checklist items: reported that a comprehensive literature search was performed, provided the characteristics of included studies, assessed and documented the scientific quality of the included studies, or appropriately addressed the quality of included studies in formulating conclusions. Less than half of reviews were compliant with the following checklist items: reported that there were duplicated study selection and data extraction, used appropriate methods to combine the findings of studies (although this varied across reviews category), or assessed the likelihood of publication bias. Few studies provided an ‘a priori’ design, reported the status of publication used as an inclusion criterion, provided a list of studies, or stated if there was a conflict of interest or not. .

## Discussion

Our study identified 369 systematic reviews of TCM interventions published since 1999, most of which evaluated herbal interventions. This study updates previous reviews of this topic [6], [7], [8] by including an additional 258 SRs. This review is also the first to examine compliance of Chinese SR authors with the PRISMA reporting guideline and AMSTAR tool for assessing methodological quality.

Our review examined some of the same variables described in Moher et al.'s investigation of SRs written in English and indexed in Medline [5]. In that study, the authors reported that the 300 SRs they included were published in 132 journals, including the Cochrane Library. In contrast to our review, only 8% of their included SRs were of complementary and alternative medicine (CAM) interventions. While most (91%) of their SRs were published in specialty journals, 45% of all 132 journals did not have an impact factor. Most (93%) of our SRs included a meta-analysis compared to 54% of the English studies and while 44% of our studies assessed for publication bias, only 23% of the English studies reporting doing so. Funding sources were reported in 39% of our studies compared to 59% of the English studies.

The range of diseases addressed in our reviews are similar with those in the three reviews [6], [7], [8] focusing on the Chinese literature. The three reviews, however, failed to report details about the epidemiological and study characteristics of systematic reviews, including number of times cited, journal type, number of studies included, number of participants, and whether it is an update of a published review. Moreover, one study [6] did not report the prevalence of reviews meeting each individual item of QUOROM. In addition we found that, despite the increasing use of the terms “systematic review” and ”meta-analysis” in the title and subsequent manuscript sections, the quality of reporting remains poor.

While many deficits in reporting were evident in the Chinese SRs, we identified areas of particular concern. These included evidence that suggested that the SRs were not highly referenced by other researchers working in the same field. This may be due, in part, to the overall poor quality of this body of work, which may also be a reason that less than one third were indexed in the Chinese Science Citation Database (CSCD), which is similar to the Science Citation Index, in that indexed journals are considered of higher quality than non-indexed journals.

There is also evidence that even though SRs have become an increasingly popular source of up-to-date knowledge, they are under utilized by Chinese clinicians. Because although the Chinese Cochrane Center was established in 1997 by the Ministry of Health of the people's republic of China, recent studies reported that most clinicians and nurses had not heard of or did not understand the meaning of evidence-based medicine (EBM) [14], [15]. In addition, not all medical schools have introduced EBM curricula in China.

Although it is well known that results from reviews are most useful when they are up-to-date [16], none of the reviews included in our SR reported being an update of a previous review. This may be due to lack of policies in China to encourage updates and reluctance of Chinese journals to publish updated reviews that are not substantially different from previous publications. The review by Moher et al. reported that 18% of the English SRs were updates, however, only those SRs published in the Cochrane Library had been updated [5], suggesting that this problem is not restricted to Chinese journals.

We have demonstrated that compliance with PRISMA reporting guidelines is low for many Chinese SRs. Items of particular concern included lack of appropriate abstracts or structured summaries and details of protocols or summaries of evidence. In order to improve the quality of reporting, we strongly recommend the use of reporting guidelines by authors. We also recommend that editors of medical journals recognize and promote use of reporting guidelines in their publications. Lastly, medical schools should introduce reporting guidelines into medical education as early as possible.

Reviews of numerous medical specialties, as well as comprehensive reviews, have concluded that the quality of SR and meta-analysis (MA) reporting is generally poor [6], [7], [17]. Examination of changes in reporting quality of MAs published in Medline, Embase and the Cochrane Database of Systematic Reviews, suggested that quality had improved after the release of the QUORUM reporting guidelines [18], [19]. We anticipate that increased use of PRISMA by Chinese authors will be helpful in increasing the reporting quality of SRs published in Chinese journals.

Our study has also shown that the methodological quality of Chinese SRs of TCM is poor. Areas of particular concern include lack of report of an ‘a priori’ design, description of status of publication used as an inclusion criterion, as well as statement of conflict of interest, all factors which may be associated with biased results. In addition, statistical mistakes appeared in one-third (29.3%) of reviews. For instance, many reviews did not explore reasons for statistical heterogeneity but simply pooled results using a random effects model to account for heterogeneity. Where heterogeneity is substantial, an overall pooling of results may be inappropriate and may lead to the incorrect interpretation of results.

One explanation for limited compliance with methodological guidelines may be that AMSTAR has not been published or promoted in Chinese journals outside of a brief abstract published in the Chinese Journal of Evidence-based Medicine in September 2010 [20]. Broader promotion of methodological quality guidelines is a necessary step in enhancing dissemination and implementation of AMSTAR.

However, our study has limitations. First, the majority of articles failed to follow the PRISMA guideline. This is likely due to the fact that the PRISMA guideline had not yet been released at the time these studies were published. Second, our study included systematic reviews published only in Chinese journals, whereas Chinese investigators increasingly publish articles in international journals. Third, we have found differential results between systematic reviews addressing herbal versus non-herbal medicines. This is possibly because reviews addressing non-herbal medicines would have studies with fewer events, resulting in absence of statistical difference of comparisons. Last, in our search, we included the terms “systematic review” and “meta-analysis”. Some potentially eligible systematic review (i.e., systematic reviews needs to be plural) may, however, not use these terms in their publications.

Our purpose was to provide readers with a broad overview of the reporting and methodological characteristics of SRs of TCM published in Chinese journals. Although many such SRs have been published, the quality of these reviews is troubling. As a potential key source of information for clinicians and researchers, not only were many of these reviews incomplete, some contained mistakes or were misleading. Focusing on improving the quality of SRs of TCM, rather than continuing to publish them in great quantity, is urgently needed in order to increase the value of these studies.

## Supporting Information

Text S1 Four Chinese databases search strategy and hyperlink address.
(DOC)

Text S2 Three hundred and sixty-nine systematic review of traditional chinese medicine interventions published in Chinese journals
(DOC)

## References

[1] Chen ZH, Zhang JH (1999). Troditional Chinese herbs for precancerous lesion of esophagus: a meta analysis.. TCM Res.

[2] Petticrew M (2000). Systematic reviews from astronomy to zoology: myths and misconceptions.. BMJ.

[3] Jadad AR, McQuay HJ (1996). Meta-analyses to evaluate analgesic interventions: a systematic qualitative review of their methodology.. J Clin Epidemiol.

[4] Shea B, Moher D, Graham I, Pham B, Tugwell P (2002). A comparison of the quality of Cochrane reviews and systematic reviews published in paper-based journals.. Eval Health Prof.

[5] Moher D, Tetzlaff J, Tricco AC, Sampson M, Altman DG (2007). Epidemiology and reporting characteristics of systematic reviews.. PLoS Med.

[6] Li TQ, Liu XM, Zhang MM, Ma JX, Du L (2007). Assessment of Systematic Reviews and Meta-analyses on Traditional Chinese Medicine Published in Chinese Journals.. Chin J Evid-based Med.

[7] Liu JP, Xia Y (2007). Quality appraisal of systematic reviews or meta-analysis on traditional Chinese medicine published in Chinese journals.. Zhongguo Zhong Xi Yi Jie He Za Zhi.

[8] ZHZANG Juhua, SHANG Hongcai, GAO Xiumei (2007). Methodology and reporting quality of systematic review/meta-analysis of traditional Chinese medicine.. J Altern Complement Med.

[9] Moher D, Liberati A, Tetzlaff J, Altman DG, PRISMA Group (2010). Preferred reporting items for systematic reviews and meta-analyses: the PRISMA statement.. Int J Surg.

[10] Shea BJ, Grimshaw JM, Wells GA, Boers M, Andersson N (2007). Development of AMSTAR: a measurement tool to assess the methodological quality of systematic reviews.. BMC Med Res Methodol.

[11] Oxman AD (1994). Checklists for review articles.. BMJ.

[12] Sacks H, Berrier J, Reitman D, Berk A, Chalmers T (1987). Meta-analyses of randomized controlled trials.. N Engl J Med.

[13] Bessa-Nogueira RV, Vasconcelos BC, Niederman R (2008). The methodological quality of systematic reviews comparing temporomandibular joint disorder surgical and non-surgical treatment.. BMC Oral Health.

[14] Wang XP, Fan GH (2010). Investigation cognition about Evidence-based Medicine.. Medical information.

[15] Liu K, Guo L, Zhang L (2010). Investigation into Nursing Staff's Cognition Status of Evidence- based Medicine.. Journal of Liao ning Medical University.

[16] Moher D, Tsertsvadze A (2006). Systematic reviews: When is an update an update?. Lancet.

[17] Moher D, Liberati A, Tetzlaff J, Altman DG, PRISMA Group (2009). Preferred reporting items for systematic reviews and meta-analyses: the PRISMA statement.. BMJ.

[18] Moher D, Cook DJ, Eastwood S, Olkin I, Rennie D (1999). Improving the quality of reports of meta-analyses of randomised controlled trials: the QUOROM statement. Quality of Reporting of Meta-analyses.. Lancet.

[19] Delaney A, Bagshaw SM, Ferland A, Manns B, Laupland KB (2005). A systematic evaluation of the quality of meta-analyses in the critical care literature.. Crit Care.

[20] Xiong J, Du YH (2010). Assessing tool of methodology quality for systematic reviews and meta analysis: introduction of AMSTAR.. Chin J Evid-based Med.

