# Supplementary material for: Epidemiology, Quality and Reporting Characteristics of Systematic Reviews of Traditional Chinese Medicine Interventions Published in Chinese Journals
Source: PLoS One. 2011 May 25;6(5):e20185. doi: 10.1371/journal.pone.0020185 (PMC3102106; doi:10.1371/journal.pone.0020185)
Supplement: Text S1 — Four Chinese databases search strategy and hyperlink address. (DOC) [file pone.0020185.s001.doc]

**Chinese Biomedicine Literature Database(CBM) search strategy:**

***Hyperlink address：***<http://sinomed.imicams.ac.cn/index.jsp>

#1 Systematic review/exp

#2 Systematic review

#3 Meta analysis/exp

#4 Meta analysis

#5 #1 or #2 or #3 or #4

#6 Traditional Chinese Medicine/exp

#7 Traditional Chinese Medicine

#8 Chinese herbs

#9 Chinese herbal drugs

#10 Traditional chinese drug

#11 acupuncture/exp

#12 acupuncture

#13 bulk herbs

#14 decoctions

#15 pills

#16 tuina

#17 massage

#18 meditation

#19 qi gong

#20 qigong

#21 #6 or #7 or #8 or #9 or #10 or #11 or #12 or #13 or #14 or #15 or #16 or #17 or #18 or #19 or #20

#22 #5 and #21

**Chinese Scientific Journal Full-text Database (CSJD) search strategy**

***Hyperlink address：***<http://www.cnki.net/>

#1 Systematic review

#2 Meta analysis

#3 #1 or #2

#4 Traditional Chinese Medicine

#5 Chinese herbs

#6 Chinese herbal drugs

#7 Traditional Chinese drug

#8 acupuncture

#9 bulk herbs

#10 decoctions

#11 pills

#12 tuina

#13 massage

#14 meditation

#15 qi gong

#16 qigong

#17 #4 or #5 or #6 or #7 or #8 or #9 or #10 or #11 or #12 or #13 or #14 or #15 or #16

#18 #3 and #17

**Chinese Journal Full-text Database (CJFD) search strategy**

***Hyperlink address：***<http://www.cqvip.com/>

#1 Systematic review

#2 Meta analysis

#3 #1 or #2

#4 Traditional Chinese Medicine

#5 Chinese herbs

#6 Chinese herbal drugs

#7 Traditional Chinese drug

#8 acupuncture

#9 bulk herbs

#10 decoctions

#11 pills

#12 tuina

#13 massage

#14 meditation

#15 qi gong

#16 qigong

#17 #4 or #5 or #6 or #7 or #8 or #9 or #10 or #11 or #12 or #13 or #14 or #15 or #16

#18 #3 and #17

**Wanfang Database search strategy**

Hyperlink address：<http://www.wanfangdata.com.cn/>

#1 Systematic review

#2 Meta analysis

#3 #1 or #2

#4 Traditional Chinese Medicine

#5 Chinese herbs

#6 Chinese herbal drugs

#7 Traditional Chinese drug

#8 acupuncture

#9 bulk herbs

#10 decoctions

#11 pills

#12 tuina

#13 massage

#14 meditation

#15 qi gong

#16 qigong

#17 #4 or #5 or #6 or #7 or #8 or #9 or #10 or #11 or #12 or #13 or #14 or #15 or #16

#18 #3 and #17
